# Examining the Relationship Between Engagement and Perceived Stress-Related Cognitive Complaints in the Argentinian Working Population

**DOI:** 10.5964/ejop.v16i1.1832

**Published:** 2020-03-03

**Authors:** Agustín Ramiro Miranda, Luisina Rivadero, Jorge Ángel Bruera, Virginia Villarreal, Laura Yhicel Bernio, Lorena de los Ángeles Baydas, Mónica Liliana Brizuela, Silvana Valeria Serra

**Affiliations:** a Escuela de Fonoaudiología, Facultad de Ciencias Médicas, Universidad Nacional de Córdoba, Córdoba, Argentina; b Instituto de Investigaciones en Ciencias de la Salud, Consejo Nacional de Investigaciones Científicas y Técnicas, Córdoba, Argentina; c Instituto de Educación Superior “Dr. Domingo Cabred”, Facultad de Educación y Salud Universidad Provincial de Córdoba, Córdoba, Argentina; d Facultad de Psicología, Universidad Nacional de Córdoba, Córdoba, Argentina; Department of Psychology and Counselling, Webster University Geneva, Geneva Switzerland

**Keywords:** occupational health, stress, executive function, memory, work engagement

## Abstract

Stress has a negative impact on cognitive functioning and occupational well-being. The aim of this study was to assess the relationship among perceived stress, cognitive complaints and work engagement in public employees from Córdoba, Argentina. In this cross-sectional study, self-report questionnaires were administered to 240 participants. Spanish versions of the following instruments were used: Perceived Stress Scale (PSS), Utrecht Work Engagement Scale (UWES), Memory Failures in Everyday (MFE), Executive Complaint Questionnaire (ECQ). Statistical analysis included ANOVA, path analysis, and multiple logistic regression. Stressed workers showed lower work engagement and more cognitive complaints, even after adjusting for demographic variables. Negative associations were also observed between work engagement and cognitive complaints, suggesting that cognitive difficulties are related to engagement. Given the relation among stress, cognition, and work engagement, it is important to consider these factors to foster workers’ health and work productivity.

In the last few years, the study of the development of positive attitudes, skills and experiences in the workplace has been addressed by occupational health psychology (Leka & Houdmont, 2010; Seligman & Csikszentmihalyi, 2000; Zoupanou & Rydstedt, 2017), hence the concept of engagement meaning a positive, persistent, emotional and cognitive state related to work (Kašpárková, Vaculík, Procházka, & Schaufeli, 2018; Orgambídez-Ramos, Borrego-Alés, & Mendoza-Sierra, 2014; Spontón, Medrano, Maffei, Spontón, & Castellano, 2012).

Kahn (1990) proposes a multidimensional approach for the definition of work engagement, defining it as the manner individuals express themselves physically, cognitively and emotionally during work roles. This conceptualization highlights three components: a physical component referring to the physical energies exerted to achieve levels of effort during work, an emotional component referring to how people feel about their work and the way in which work meets their emotional demands, and a cognitive component referring to the mental processes underlying the work role, such as attention, vigilance and concentration. In addition, Kahn (1990) identifies three psychological conditions that have an impact on work engagement: psychological fullness (i.e. rewarding idea by being intensely involved in work), psychological security (i.e. getting involved in work without suffering negative consequences for self-image or status), and psychological availability (i.e. the sense of having the necessary personal resources to perform the task favourably).

The conceptualization made by Kahn (1990) was later recovered by Rothbard (2001), who defines engagement as the psychological state associated with activities linked to the work role. Rothbard (2001) suggests that engagement is determined by two critical components: attention and absorption. In this two-dimensional motivational construct, absorption refers to the intensity of immersion individuals experience at work, and attention refers to the cognitive resources, including concentration and psychical energy that individuals invest during work. Later, Rich, Lepine, and Crawford (2010) expanded the concept and introduced subtle changes. Authors integrated Kahn’s (1990) three-dimensional concept and Rothbard’s (2001) two components into Brown and Leigh (1996) and Russell and Barrett (1999) investigations. The study of Brown and Leigh (1996) was retrieved to evaluate the physical aspect of engagement, whereas the study of Russell and Barrett (1999) was used to define the emotional component of engagement.

Conversely, several authors use the concept of burnout to define, by opposition, the concept of engagement. According to Maslach and Leiter (1997), engagement can be conceptualized as the opposite pattern to burnout. In this sense, engagement represents a state of high energy levels, participation and effectiveness, and burnout represents exhaustion, cynicism and inefficiency. Nevertheless, Schaufeli, Salanova, González-Romá, and Bakker (2002) define burnout and engagement as two distinct constructs that must be accessed independently. From this perspective, if an employee does not experience burnout, he or she should not necessarily be affected by engagement, and *vice versa*. This theorization gives rise to one of the most widespread definitions of engagement: "... a positive, satisfactory and work-related mental state characterized by vigour, dedication and absorption" (Schaufeli et al., 2002, p. 74). These dimensions were operationalized by the theorists with the development of the Utrecht Work Engagement Scale, an instrument designed to measure work engagement. Vigour is related to high energy levels and the desire to invest effort and endeavour in work. Dedication is associated with involvement levels and meaning of work. Absorption refers to complete concentration and enjoyment at work (Bakker, 2011).

Engagement is intimately linked to the conservation of resources theory (Hobfoll, 1989), an integrated model that incorporates several approaches to understand the effects of stress. According to this theory, people tend to make efforts to achieve to obtain, retain, protect and create resources important to them. Resources can be either material (e.g., food), personal (e.g., self-esteem), living conditions (e.g., family support), and energy resources (e.g., cognitive status). When these resources are in danger, lost or not obtained after investing in them, subjects begin to experience stress. Therefore, the availability of these resources determines the efficiency with which people can adapt to the demands underling their daily life, such as those required to succeed in their work role.

Meijman and Mulder (1998) developed the effort-recovery theory, which establishes that continuous exposure to stressful demands (related or not related to work) poses a risk to employees’ optimal functioning, since stressful demands compromise the restoration of energy resources and self-regulation mechanisms, in accordance to Baumeister’s (1998) self-regulation theory. In this sense, self-regulatory capacity is essential for work engagement, which is achieving a state of cognitive, affective and behavioural connection with work.

Moreover, engagement has been consistently associated with work performance (Halbesleben & Wheeler, 2008), as well as with workers’ psychological and physical health (Hamilton Skurak, Malinen, Näswall, & Kuntz, 2018). In this regard, it is related to higher cognitive and behavioural performance, and lower depression risk. Schaufeli and Bakker (2004) observed positive cognitive and behavioural effects of engagement, such as work satisfaction and proactive performance. What is more, lower stress, depression levels (Schaufeli, Bakker, & Van Rhenen, 2009) and psychosomatic complaints (Demerouti, Bakker, Nachreiner, & Schaufeli, 2001) were reported in engaged workers. Therefore, to foster occupational health it is essential not only to develop healthy workplaces and labour practices, but also to identify conditioning factors for engagement, among which stress highlights. In this vein, workers who experience lower levels of stress might have higher levels of engagement (Anthony‐McMann, Ellinger, Astakhova, & Halbesleben, 2017).

As employees must have the capacity (both environmental and personal) to successfully invest in their engagement while facing daily conflicts, they require great material, social, personal and energy resources (Folkman, 2011). In the face of high daily-life stressful demands, employees reduce their engagement levels as they prioritize everyday-life demands. This also prevents the depletion of resources for potential future demands. As a result, gains and losses cycles are generated (Hakanen, Perhoniemi, & Toppinen-Tanner, 2008). Losses spirals indicate those employees with reduced resources are more vulnerable to suffer future resource losses. On the contrary, gains spirals indicate that employees who obtain more resources are more capable of gaining resources, resulting in future gains. In consequence, “as people make resource gains and successfully experience the rewards of dedication and absorption, they experience more positive health and well-being and become more capable of further investing resources into the engagement process” (Hobfoll, 2011, p. 138).

Concerning cognitive complaints, studies account for an association with stress. Workers with chronic stress might suffer cognitive impairments due to hippocampus damage (Barbe, Kimble, & Rubenstein, 2018). Moreover, prolonged work-related stress has a negative impact on attention, learning, and memory domains (Eskildsen et al., 2017), as on concentration, information processing (Elfering, Grebner, & Dudan, 2011), and decision-making abilities (Ceschi, Demerouti, Sartori, & Weller, 2017). In this sense, there are several studies describing the association between cognitive deficits and work performance (Birnbaum et al., 2010; McIntyre et al., 2015). In clinical populations, such as patients with major depressive disorder, cognitive impairments in the domains of executive functioning influence work productivity (Clark, DiBenedetti, & Perez, 2016). Executive functions are higher-order cognitive processes, which form a construct composed of multiple interrelated skills that serve goal-directed behaviour (Miranda, Franchetto Sierra, et al., 2019). Hence, these processes have significant implications for work.

Given the evidence accounting for the benefits, reliability and validity of self-report instruments, a growing number of health-related research use this approach (Gharibi et al., 2016; Gordts, Uzieblo, Neumann, Van den Bussche, & Rossi, 2017). Barbe et al. (2018) found a relationship among subjective cognitive complaints, perceived stress, and work performance in healthcare workers by using self-report measures. Although engagement is a crucial factor in human-services occupations (educators, health workers and customer service workers) for its positive outcomes related to client satisfaction and organizational performance (Bakker, Demerouti, & Sanz-Vergel, 2014), there lacks evidence on its association with stress and cognition in this population. Based on the above arguments and theories, we hypothesize that perceived stress is negatively associated with the level of work engagement, being mediated by cognitive-perceived dysfunctions. Thus, the present study was conducted with the aim of assessing the relationship among perceived stress, cognitive complaints and work engagement among public employees from Córdoba, Argentina.

## Method

### Subjects

In this cross-sectional study, self-report questionnaires were administered to a total of 286 public employees from Córdoba (Argentina) that participated in one of the two editions of a Short-Term Voice Training Course in 2018, conducted by the School of Speech and Language at the National University of Córdoba in collaboration with Government of the Province of Córdoba, Argentina. 240 participants returned the questionnaires after the training course (questionnaire return rate: 83.91%). The majority of participants in the sample were women (86%). Sixty-seven percent were in couple (married or living with a partner). Most of the sample (96%) reported an education of 12 years or more. Eighty percent of the subjects did not report medical diseases. Participants’ occupational characteristics (occupation, hours/day, years of service and working shift) are summarized in Table 1.

**Table 1:** Demographic Characteristics of 240 Public Employees From Córdoba (Argentina, Year 2018)

Variable	*N*	%
Age, *M* (*SE*) = 43.18 (0.59)		
24-36	71	29.58
37-49	105	43.75
50-63	63	26.25
Sex		
Women	206	85.83
Men	34	14.16
Educational level		
< 12 years of instruction	9	3.75
≥ 12 years of instruction	231	96.25
Occupation		
Teaching work	172	71.66
Administrative work	57	23.75
Other	11	4.58
Hours/day		
≤ 4	34	14.17
5-9	179	74.58
10-14	26	10.83
≥ 15	0	0.00
Years of service		
≤ 4	35	14.58
5-9	42	17.50
10-14	42	17.50
15-19	37	15.42
20-24	33	13.75
25-29	27	11.25
≥ 30	24	10.00
Working shift		
Morning	105	43.75
Evening	52	21.66
Morning-evening	52	21.66
Night	3	1.25
Rotating	28	11.66
Marital status		
Single	79	32.92
In couple	161	67.08
Medical disease		
No	193	80.42
Yes	47	19.58
Perceived stress category		
Low	54	22.50
Moderate	115	47.92
High	71	29.58

### Instruments

#### Engagement

Was assessed by using the Spanish version of Utrecht Work Engagement Scale (UWES) (Spontón et al., 2012). This version consists of 17 items grouped into three subscales, as revealed by the factor analysis: “vigour” (Items 1, 4, 8, 12, 15, 17; e.g.: “At my work I always persevere, even when things do not go well”), “dedication” (Items 2, 5, 7, 10, 13; e.g.: “I find the work that I do full of meaning and purpose”), and “absorption” (Items 3, 6, 9, 11, 14, 16; e.g.: “When I am working, I forget everything else around me”). Each item was rated on a 7-point Likert´s scale (0 to 6, where 0 indicates *never* and 6 corresponds to *everyday*). Scores were transformed on a scale ranging from 0 to 102, where higher scores indicate higher work engagement. Previous studies have tested the psychometric properties of the UWES, showing its good construct validity and reliability (Schaufeli, Salanova, González-Romá, & Bakker, 2002; Spontón et al., 2012). In this study, Cronbach’s coefficient α were calculated to assess internal consistency reliability, showing acceptable values ranging from .82 to .94 (Table 2).

#### Cognitive Complaints

Two self-report questionnaires were applied. First, participants responded to a Spanish modified version of Memory Failures of Everyday (MFE) questionnaire, which explores subjective memory complaints and related cognitive processes, such as attention, perceptual recognition, and language (Lozoya-Delgado, de León Ruiz-Sánchez, & Pedrero-Perez, 2012). The Spanish version of MFE consists of 30 items (e.g.: “Forgetting important details of what happened to you the day before” and “Having difficulty picking up a new skill”) and was designed in a 5-point Likert's scale (0 to 4, where 0 indicates *never* and 4 corresponds to *always*). Psychometric analyses of this questionnaire showed it is unifactorial and reliable (García Martínez & Sánchez Cánovas, 1994).

In addition, the Executive Complaint Questionnaire (ECQ) was used (Mías, 2010). This questionnaire explores the subjective complaints of executive functions. To respond the questionnaire, the subject must judge to what extent a series of behaviours of daily life concerning mental functioning in general occur. The questionnaire has 15 items divided into five subscales that assess the presence of cognitive, behavioural and emotional symptoms related to the functioning of the prefrontal cortex. Each item was rated on a 5-point Likert´s scale (0 to 4, where 0 indicates *never* and 4 corresponds to *always*). The original factor analysis showed the following subscales: “attention and immediate memory” (Items 1, 6 and 11; e.g.: “You find it difficult to recall messages after a few minutes”), “initiative and scheduling” (Items 2, 7 and 12; e.g.: “You need to be pushed into action to start some activity off”), “behavioural flexibility” (Items 3, 8 and 13; e.g.: “You find it difficult to adapt to new situations”), “apathy and decision” (Items 4, 9 and 14; e.g.: “You find it difficult to take an interest in new things”), and “inhibitory control” (Items 5, 10 and 15; e.g.: “Your mood might abruptly and remarkably change”). Scores of each subscale are made up by the sum of the three corresponding items divided by four, with values ranging from 0 to 4 (higher scores indicate higher degree of executive complaints). Both questionnaires have recently been validated in the general Spanish-speaking population showing adequate psychometric properties (Miranda, Rivadero, Serra, & Soria, 2019; Lozoya-Delgado et al., 2012).

#### Perceived Stress

The Spanish version of Perceived Stress Scale (PSS-10) was used (Campo-Arias, Bustos-Leiton, & Romero-Chaparro, 2009). This 10 item self-report scale evaluates the level of perceived stress during the last month (e.g.: “In the last month, how often have you been/felt unable to control the important things in your life?” and “In the last month, how often have you been/felt confident about your ability to handle your personal problems?”). The items are rated on a 5-point Likert’s scale (0 to 4, where 0 indicates *never* and 4 corresponds to *very frequently*). Scores of Items 4, 5, 6, 7, 9, 10, and 13 are reversed. Higher scores correspond to higher perceived stress.

Internal consistency (α) for the four scales and subscales are presented in Table 2. The α for PSS-10, MFE-30, UWES, ECQ were found to be .78, .95, .94, .90, respectively, indicating satisfactory results (Tavakol & Dennick, 2011). Regarding UWES subscales, “vigour”, “dedication” and “absorption” revealed being adequate. Acceptable values were found for the majority of ECQ-subscales, except for “inhibitory control” (α = .58).

**Table 2:** Internal Consistency Reliability of the Measuring Instruments

Instrument	Cronbach’s α	Number of items
PSS-10	.78	10
MFE-30	.95	30
UWES	.94	17
Vigour	.85	6
Dedication	.91	5
Absorption	.82	6
ECQ	.90	15
Attention and immediate memory	.79	3
Initiative and scheduling	.74	3
Behavioural flexibility	.71	3
Apathy and decision	.60	3
Inhibitory control	.58	3

This research was in accordance with Declaration of Helsinki and current legislation, being approved by the corresponding Ethics Committee of the National University of Córdoba (registration: RePIS-3450 - Córdoba). For all participants who chose to enrol in the study, informed consent was obtained, and their responses were confidential and anonymous.

### Statistical Analysis

All statistical analyses were performed using the InfoStat software (version 2012, Infostat Group, Argentina), except for the internal consistency analysis that was performed by IBM SPSS Statistics (version 22.0; IBM Inc., USA). *P* values below .05 were considered significant (*p* < .05).

The reliability related to internal consistency was measured by α coefficient. Descriptive statistics (mean, standard error, median and range) were calculated with all variables; percentage of sex category was also described. Analysis of variance (ANOVA, followed by Tukey test) and Student's *t* test were used to estimate differences among sociodemographic and occupational groups (Table 1), as well as perceived stress groups. Based on the approach of Othman, Ahmad, El Morr, and Ritvo (2019), participants were divided into three categories according to their PSS sum scores: low stress (from 0-13), moderate stress (from 14-26), and high stress (from 27-40).

The path coefficient analysis method was used to identify the possible causal explanations of the correlations observed between engagement (response variable) and cognitive complaints and perceived stress (predictors) (Balzarini et al., 2015; Valencia-Ramírez & Ligarreto-Moreno, 2012). In other words, this method allows to decompose the correlation between a variable X and the final product (Y), in a "direct" path of X over Y and in "indirect" path of X over Y, which are made effective through the relationship of X with other components of Y (Z, W, etc.) (Di Rienzo et al., 2016). The following variables were considered as component X: executive complaints (“attention and immediate memory”, “initiative and scheduling”, “behavioural flexibility”, “apathy and decision”, and “inhibitory control”), memory complaints, and perceived stress. The following variables were considered as component Y: overall engagement, vigour, absorption and dedication. Multiple logistic regression analysis was used to estimate the adjusted odds ratio (AOR) of each of the executive complaint according to perceived stress, comparing the high-stressed employees with the low and moderate perceived stress groups as the reference category and simultaneously controlling for age, sex, educational level, and medical disease.

## Results

Descriptive statistics of questionnaires scores are presented in Table 3. By comparing scores between groups according to demographic and occupational variables, significant differences using test T were only found for sex and medical disease. In this sense, women showed higher scores in PSS-10 (15.23 vs. 12.47; *p* = .0026) and “vigour” subscale (27.88 vs. 24.71; *p* = .0011) than men. Healthy subjects reported lower “apathy and decision” (0.88 vs. 1.14; *p* = .04) and “behavioural flexibility” (1.13 vs. 1.49; *p* = .003) scores.

**Table 3:** Summary Table of the Mean, Standard Error, Minimum, Maximum and Median Values of Questionnaires Scores of 240 Public Employees From Córdoba (Argentina, Year 2018)

Instrument	*M* (*SE*)	Min-Max	Median
PSS-10	14.81 (0.32)	3-31	15
MFE-30	20.70 (1.56)	0-107	18
UWES	81.54 (1.10)	3-102	85
Vigour	29.34 (0.37)	1-36	30
Dedication	25.06 (0.36)	1-30	27
Absorption	27.38 (0.43)	1-36	29
ECQ	17.85 (0.57)	0-49	17
Attention and immediate memory	1.32 (0.05)	0-3.67	1.33
Initiative and scheduling	1.07 (0.05)	0-4	1
Behavioural flexibility	1.20 (0.05)	0-3.67	1.33
Apathy and decision	0.93 (0.04)	0-4	1
Inhibitory control	1.42 (0.05)	0-4	1.33

**Figure 1:**
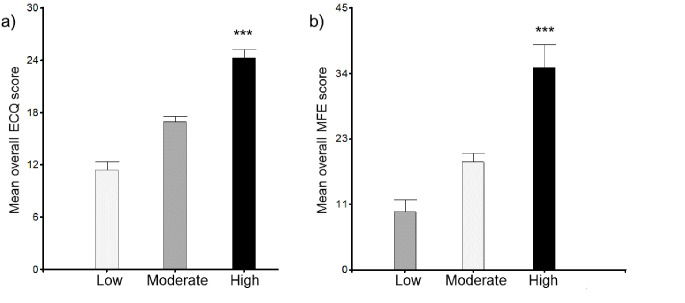
Overall scores of the Executive Complaints Questionnaire (a) and the Memory Failures in Everyday (b) by perceived stress groups (Low *n* = 54; Moderate *n* = 115; High *n* = 71). Statistical analysis was performed using ANOVA with Tukey test. Error bars represent the standard error of the mean. Higher scores of ECQ and MFE indicate a higher frequency of self-reported cognitive failures. ***p < .0001.

**Figure 2:**
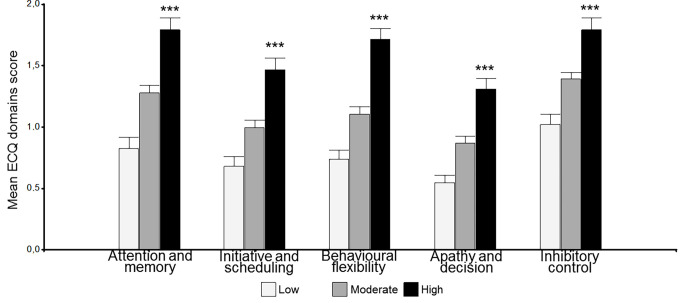
Executive Complaints Questionnaire scores by perceived stress groups (Low *n* = 54; Moderate *n* = 115; High *n* = 71). Statistical analysis was performed using ANOVA with Tukey test. Error bars represent the standard error of the mean. Higher scores indicate a higher frequency of self-reported executive failures. ***p < .0001.

When subjects were classified into stress groups based on perceived stress, levels of executive complaints were significantly higher in the high group, 24.22 (0.90), than in the low, 11.40 (1.00), and moderate, 16.90 (0.69), groups (Figure 1 a). Moreover, when analysing ECQ domains, this significant difference was also present in the five subscales (Figure 2). Similarly, differences were found between groups in memory complaints: low group, 9.94 (2.87), moderate group, 18.48 (1.90), and high group, 34.74 (2.70), *p* < .0001 (Figure 1 b).

**Figure 3:**
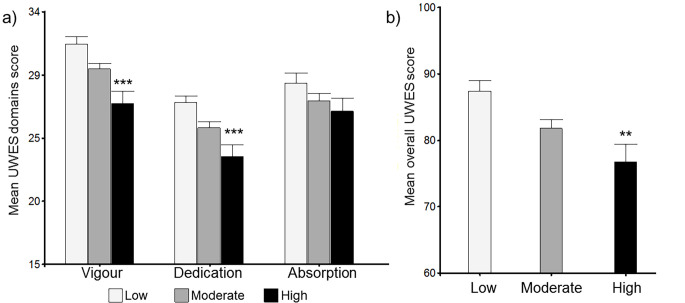
Overall and domains scores of the Utrecht Work Engagement Scale by perceived stress groups (Low *n* = 54; Moderate *n* = 115; High *n* = 71). Statistical analysis was performed using ANOVA with Tukey test. Error bars represent the standard error of the mean. Higher scores indicate a higher work engagement. **p < .001. ***p < .0001.

In addition, the group with higher perceived stress reported lower scores in engagement (Figure 3). Mean scores of overall UWES were 87.42 (2.28) for the low group, 81.78 (1.59) for the moderate group and 76.77 (1.96) for the high group, being significantly different (*p* = .002). Further, lower levels of “vigour” and “dedication”, but not “absorption”, were found in subjects with low and moderate perceived stress.

On the other hand, it was found that perceived stress correlated significantly with “vigour” (path coefficient = -.42, *p* < .0001), “dedication” (path coefficient = -.39, *p* < .0001) and “absorption” (path coefficient = -.26, *p* < .001). These correlations have shown to be mainly determined by the correlation between these three occupational variables with “attention and immediate memory” (path coefficients = -.27, -.22, -.19, respectively) and “inhibitory control” (path coefficients = -.20, -.20, -.21, respectively). Similarly, the correlation between engagement and perceived stress was significant (path coefficient = -.37, *p* < .0001). In this case, there was a greater participation of “attention and immediate memory” (path coefficient = -.24) and “inhibitory control” (path coefficient = -.22). The direct effect of stress was low (path coefficients between -.01 and -.05). When analysing the effects of cognitive complaints on work engagement variables, perceived stress was found to participate indirectly in the associations, showing negative correlations and explaining moderately the total effect. Table 4 presents the estimates of path coefficients analysis.

**Table 4:** Path Coefficients of Direct and Indirect Effects of Cognitive Complaints and Perceived Stress on Work Engagement

Mediator variable models	AIM	IS	BF	AD	IC	MFE	PSS
Vigour (*p* < .0001; CI [33.83-40.02])							
Attention and immediate memory (AIM)	**.29**	-.16	.13	-.38	-.09	-.08	-.27
Initiative and scheduling (IS)	-.10	**.18**	.16	.14	.13	.20	.15
Behavioural flexibility (BF)	.08	.09	**-.11**	-.10	-.08	-.10	-.08
Apathy and decision (AD)	-.18	-.23	-.26	**.09**	.07	.07	.07
Inhibitory control (IC)	-.04	-.05	-.05	-.04	**-.18**	-.21	-.20
Memory Failures in Everyday (MFE)	-.05	-.05	-.04	-.04	-.04	**-.04**	-.04
Perceived Stress Scale (PSS)	-.14	-.14	-.14	-.15	-.13	-.16	**-.05**
Total effect	-.16	-.36	-.31	-.48	-.32	-.32	-.42
*p*-value	n/s	***	***	***	***	***	***
Dedication (*p* < .0001; CI [28.93-34.45])							
Attention and immediate memory (AIM)	**.18**	-.06	.11	-.38	-.13	-.05	-.22
Initiative and scheduling (IS)	-.04	**.11**	.10	.09	.08	.12	.10
Behavioural flexibility (BF)	.06	.08	**-.04**	-.03	-.03	-.04	-.03
Apathy and decision (AD)	-.19	-.23	-.26	**.07**	.06	.06	.06
Inhibitory control (IC)	-.06	-.07	-.07	-.06	**-.18**	-.21	-.20
Memory Failures in Everyday (MFE)	-.03	-.03	-.02	-.02	-.02	**-.06**	-.06
Perceived Stress Scale (PSS)	-.12	-.11	-.12	-.12	-.10	-.13	**-.03**
Total effect	-.19	-.31	-.30	-.46	-.33	-.30	-.39
*p*-value	*	***	***	***	***	***	***
Absorption (*p* < .0001; CI [29.87-37.10])							
Attention and immediate memory (AIM)	**.35**	-.16	.26	-.39	-.17	-.02	-.19
Initiative and scheduling (IS)	-.10	**.21**	.20	.17	.16	.24	.18
Behavioural flexibility (BF)	.15	.18	**-.11**	-.10	-.08	-.10	-.08
Apathy and decision (AD)	-.19	-.24	-.27	**.17**	.13	.14	.14
Inhibitory control (IC)	-.08	-.08	-.09	-.08	**-.19**	-.21	-.21
Memory Failures in Everyday (MFE)	-.02	-.01	-.01	-.01	-.01	**-.08**	-.08
Perceived Stress Scale (PSS)	-.10	-.10	-.10	-.10	-.09	-.11	**-.01**
Total effect	.01	-.20	-.13	-.35	-.24	-.15	-.26
*p*-value	n/s	*	n/s	***	**	n/s	**
Total engagement (*p* < .0001; CI [93.24-110.98])							
Attention and immediate memory (AIM)	**.29**	-.14	.18	-.41	-.14	-.05	-.24
Initiative and scheduling (IS)	-.09	**.18**	.17	.14	.14	.20	.15
Behavioural flexibility (BF)	.10	.13	**-.10**	-.08	-.07	-.09	-.07
Apathy and decision (AD)	-.20	-.25	-.27	**.12**	.09	.10	.10
Inhibitory control (IC)	-.06	-.07	-.07	-.07	**-.19**	-.22	-.22
Memory Failures in Everyday (MFE)	-.03	-.03	-.03	-.03	-.02	**-.07**	-.06
Perceived Stress Scale (PSS)	-.13	-.12	-.13	-.13	-.11	-.14	**-.03**
Total effect	-.11	-.30	-.25	-.45	-.31	-.27	-.37
*p*-value	n/s	***	***	***	***	**	***

*Note.* Direct effects in bold. CI = confidence interval.

*p < .05. **p < .001. ***p < .0001.

The AOR for executive complaints according to perceived stress category are shown in Table 5. These estimates were obtained from the multiple logistic regression model with low/moderate stress as the reference category and controlling for age, sex, educational level, and medical disease. As shown, the AOR of executive complaints increased as perceived stress increased. When compared to the reference category, subjects with high perceived stress were 7.78 times more likely to report greater executive complaints (AOR = 7.78, 95% CI [3.86, 15.69]. Moreover, perceived stress associated positively with executive domains. High-stressed participants were significantly more likely to report complaints in “attention and immediate memory” (AOR = 5.29, 95% CI [2.64, 10.63], “initiative and scheduling” (AOR = 3.35, 95% CI [1.84, 6.11], “behavioural flexibility” (AOR = 6.04, 95% CI [3.05, 11.98], “apathy and decision” (AOR = 4.09, 95% CI [2.13, 7.85], and “inhibitory control” (AOR = 3.09, 95% CI [1.68, 5.68].

**Table 5:** Adjusted Odds Ratios (AOR) for Executive Complaints Depending on Perceived Stress

Executive function domain	AOR^a^	95% CI	*p*
Attention and immediate memory	5.29	[2.64, 10.63]	< .0001
Initiative and scheduling	3.35	[1.84, 6.11]	.0001
Behavioural flexibility	6.04	[3.05, 11.98]	< .0001
Apathy and decision	4.09	[2.13, 7.85]	< .0001
Inhibitory control	3.09	[1.68, 5.68]	.0003
Overall executive complaints	7.78	[3.86, 15.69]	< .0001

*Note.* CI = confidence interval.

^a^Adjusted for age, sex, educational level, and medical disease. High-stressed employees compared with low/moderate-stressed ones (reference category).

## Discussion

The aim of this study was to assess the relationship among perceived stress, cognitive complaints and work engagement among public employees from Córdoba, Argentina. According to the study results, stressed workers showed lower work engagement and more cognitive complaints.

First, regarding the relation between stress and cognitive complaints, our results are consistent with previous cross-sectional findings. Oosterholt, Maes, Van der Linden, Verbraak, and Kompier (2014) showed that stressed workers have more cognitive problems than non-stressed workers by evaluating cognitive performance using self-report questionnaires and neuropsychological tests. Accordingly, Gavelin, Boraxbekk, Stenlund, Järvholm, and Neely (2015) showed a direct correlation among stress and cognitive impairments, especially in executive, attentional, and mnesic domains. This might be explained by the deleterious effect that stress-related hormones have on the prefrontal system and the medial temporal lobe (Alderson & Novack, 2002; Lupien & Lepage, 2001; Öhman, Nordin, Bergdahl, Slunga Birgander, & Stigsdotter Neely, 2007). Executive impairments might be due to changes in the employees’ prefrontal system (Öhman et al., 2007), for it is involved in cognitive processes such as “attention and immediate memory”, “initiative and scheduling”, “behavioural flexibility”, “apathy and decision”, and “inhibitory control”. In the same vein, chronic stress remodels hippocampus neural architecture, leading to memory impairments (McEwen, Nasca, & Gray, 2016).

Moreover, negative associations were observed between work engagement and cognitive complaints, suggesting that cognitive difficulties have an impact on engagement. All the theories proposed to explain engagement define it as a multidimensional concept (Eldor & Vigoda-Gadot, 2017). Among the most important theoretical models is the one proposed by Rothbard (2001), which theorizes that two critical components are involved in engagement: attention and absorption. Both components are determined by the employees’ cognitive status, since subjects require cognitive energy to be deeply engrossed and not easily distracted, to ignore distractors, concentrate, and inhibit behaviour. Subsequently, Rich et al. (2010) propose a three-dimensional model, describing physical, based on the theory of Brown and Leigh (1996), and emotional, based on Russell and Barrett (1999), components in addition to the cognitive components. The three-dimension approach used in this study, proposed by Schaufeli and Bakker (2010), suggests that engaged workers require cognitive energy to invest in their work practice.

We detected through path analysis that the effect of cognitive complaints on engagement was specifically mediated by concerns about “attention and immediate memory” and “inhibitory control”. In this regard, Barbe et al. (2018) found that perceived stress and subjective cognitive complaints were associated with lower work performance. What is more, cognitive impairments may affect work safety (Fisher, Chaffee, Tetrick, Davalos, & Potter, 2017). In accordance with other study, women reported higher levels of stress, which could be due to the presence of more stressful life events (Cohen & Janicki‐Deverts, 2012). In addition, there exist evidence supporting the fact that workers with cognitive difficulties show less vigour and dedication (Fisher et al., 2017). The compromise of resources in stressed workers has an impact on cognitive abilities during work performance (Ford, Cerasoli, Higgins, & Decesare, 2011). This might explain the lack of engagement in employees who report cognitive complaints, since cognition is essential to learn and develop work-related skills (Salthouse, 2012). In line with our results showing that executive functioning is associated with low work engagement, Fisher et al. (2017) suggested that poor inhibitory control affects learning processes by draining already limited resources. Moreover, the link between stress and engagement might be modulated by the cognitive status (Deligkaris, Panagopoulou, Montgomery, & Masoura, 2014), which was also demonstrated by our results through the path analysis: concerns in “inhibitory control” and “attention and memory” explained the indirect effect of perceived stress on work engagement.

On the other hand, the results of this study indicated a negative association between perceived stress and engagement. Stressed individuals showed lower “vigour” and “dedication”. This finding is in line with self-regulatory, effort-recovery, and conservation of resources theories (Barber, Grawitch, & Munz, 2013; Hobfoll, 1989). In this sense, chronic stress leads to poor self-regulation of resources needed for work engagement maintenance. Therefore, employees with higher stress levels undergo lower energy levels and are less enthusiastic about work related tasks. In consequence, work performance is compromised (Guerrero & Puerto Barrios, 2007). Finally, similar internal consistency values to those reported previously were recorded in this investigation for all the self-report questionnaires used (González-Ramírez, Rodríguez-Ayán, & Hernández, 2013; Lozoya-Delgado et al., 2012; Rodríguez Montalbán, Martínez-Lugo, & Sánchez-Cardona, 2014).

Some limitations of our study should be addressed. First, although we had adjusted for a variety of individual characteristics and used different statistical approaches, the cross-sectional nature limits the inference on causality among variables (Rothman & Greenland, 2005). In spite of having used path analysis to assess our hypothesized model to investigate whether data were consistent with causal links between outcome measures and predictors, cause-and-effect relationship cannot be established. Thus, the present findings contribute to available evidence, and open new questions for further research using longitudinal designs. Second, since the sample size may be considered a limitation, we suggest conducting studies with larger samples. Third, 85% of the participants were women, a higher sex ratio than the reported in Argentina (51.33% of women) (National Institute of Statistics and Censuses, 2010). However, it should be noted that there is a predominance of women in the education field (Ferreira et al., 2010), which indicates that the study sample is representative of that population, justifying the scope of this study. Fourth, concerning the sample selection, participants were recruited from a voice-training course, which may have influenced the composition and characteristics of the sample, limiting generalizability (Giannini, Latorre, Fischer, Ghirardi, & Ferreira, 2015).

Results discussed in this article allow a better understanding of mental health processes related to work engagement. Briefly, given the association between stress, cognition, and work engagement, it is important to consider these factors to foster workers’ health and work productivity. Neuropsychological difficulties might affect the cognitive resources necessary to perform the tasks and functions required at the workplace. Therefore, from a paradigm based on the conservation of resources theory, policies and interventions should be oriented towards the prevention of stress, the identification of cognitive and mental complaints, and the promotion of engagement. Considering the results published by Knight, Patterson, and Dawson (2017), the creation of labour resources, creation of personal resources, training for leadership and health promotion are common interventions to promote work engagement. Thus, future investigations should be oriented towards the evaluation of the impact of these interventions in different work environments.

**Ethical Approval**

This study was approved by the corresponding Ethics Committee of the National University of Córdoba (registration: RePIS-3450 - Córdoba). All procedures performed were in accordance with the Helsinki declaration.
